# Clinical Demonstration of Selected Skin Cases

**Published:** 1909-06-26

**Authors:** G. Norman Meachen

**Affiliations:** Physician to the Skin Department, Prince of Wales's General Hospital


### Hospital Clinics.

### CLINICAL DEMONSTRATION OF SELECTED SKIN CASES.

By G. NORMAN MEACHEN, M.D., B.S., M.R.C.P.(Lond.), Physician to the Skin Department, Prince of Wales's General Hospital.

(Given at the North-East London Post-Graduate College).

### Symmetrical Comedones.

Gentlemen,—This little boy, aged two, has had these curious symmetrical groups of comedones upon his face for the last eight months. You can see them scattered in groups upon the forehead, cheeks, and chin; the chest and back are free, and there are no papules or pustules anywhere. Careful questioning of the mother has failed to elicit the fact of any irritating application having been made to the child's skin, for, as you know, it is usually regarded as more or less of an axiom that these lesions in children are always produced by some application. Since their appearance she has been using several of the widely advertised proprietary remedies which profess to "cure all skin diseases." One text-book upon the subject says that comedones never occur in young children, but in the case before us we have an exception to this statement, and, although rare, they are not so excessively uncommon. The condition is easily cured by gentle friction with a little soft soap, followed by the inunction of a mild sulphur ointment.

### Epithelioma following Lupus.

In this old lady, aged 76, we have an example of, perhaps, the most terrible of the complications of lupus, epithelioma. She was sent up to the hospital by Dr. Best, of Cheshunt, with the history that her face has been "rough," *i.e.* both hirsute and affected with lupus, for the last twenty years. During most of this period she has been compelled, for the sake of appearances, to shave the hair, and you can see now that were this not done she would have quite a respectable growth of hair upon the chin and sides of the face. There is no family history of phthisis. Four months ago a "rough sore" was noticed by her which has gradually spread up to the present time; now over the right malar region, encroaching upon the outer canthus, there is an ulcerated surface with indurated, heaped-up margins of a very formidable appearance. There are no enlarged glands. The lupoid area, which is practically quiescent, extends over the whole of the right side of the face and ear, with a spur across the nose. The patient's general health is good.

The number of cases of lupus in which epithelioma develops is about 2 per cent., but Dubois-Havenith has met with it in as many as five out of his 118 cases. A few cases have developed while patients have been under treatment for their lupus by *x*-rays, such cases having been described by Norman Walker, Da Costa, and others; but it should be clearly understood that any irritant, whether *x*-rays, the act of scratching, or the process of shaving, as in the present case, may, under suitable circumstances and at this period of life, favour the development of epithelioma or carcinoma cutis upon the lupoid area. Considering the close proximity of the orbit to the growth, I fear that surgical measures are out of the question. She has therefore had, at my request, a very mild exposure to the rays, and, failing any beneficial effect from them, I propose to apply a caustic to the base of the ulcer.

### Two Cases of Lupus Erythematosus.

The next case, a young single woman, aged 35, has extensive lupus erythematosus of the face and ears. She has suffered for three years from the disease, and she also gets chilblains in the cold weather. One brother died of consumption. She has not quite the typical "butterfly-patch" of the disease, but rather disseminated areas involving the cheeks, forehead, scalp, and nose. Comparing her present condition with this photograph, taken six weeks ago, I think you will agree that there is considerable improvement. The treatment has consisted of a weekly painting of the affected areas with a mixture of equal parts of powdered camphor and pure phenol, and the internal administration of salicin in full doses, ten grains three times a day. We tried her with quinine at first, after Payne's method, but she suffered too severely from headache to allow of its continuance. The relation of lupus erythematosus to tuberculosis is most interesting. Boeck's statistics show that 28 out of 42 cases presented undoubted evidences of tuberculosis. Sequeira's figures at the London Hospital show that 34 out of 71 cases gave a marked family history of tuberculosis.

I have a unique case here of the same affection involving the scalp only in a married woman of 59. It is very rare to get lupus erythematosus affecting the scalp alone. This patient has suffered for six years. A case of a similar nature was exhibited by Stowers before the Dermatological Society of Great Britain and Ireland in 1898 in which the scalp only had been affected for eleven years. This patient suffers much from headache and a burning sensation in the scalp. You will see how extensive the disease is here, involving the whole of the occipital and a portion of the left parietal area, which is completely denuded of hair, and presents the characteristic circumscribed, reddened patches, slightly scaly and rough, the surrounding parts being perfectly smooth and atrophic. In her case there is the most remarkable history of her having lost eight children with tuberculosis of various organs! I much prefer Unna's name of "ulerythema centrifugum" for this affection. It describes accurately the clinical features —a "scar-leaving erythema, fleeing from the centre"—and, moreover, it has the great advantage of not frightening the patient, for the mere mention of the word "lupus" is often a great alarm to nervous individuals, and naturally so.

### Eczema and Xerodermia.

This little boy, aged five, is now recovering from an eczema engrafted upon a xerodermic basis. His skin, as you can see well upon the shins and face, is harsh, dry, and the natural lines are much exaggerated. The condition is said to have manifested itself the second year after birth, though, in all probability, it was really congenital. One aunt suffers also from a "very dry skin." Such skins are very prone to get eczema of the chapped and fissured variety—eczema rimosum—and this little fellow's palms and soles were severely affected a few weeks ago. I have ordered an ointment composed of glyc. amyli dr. 2, ung. glyc. plumbi subacet. dr. 2, lanoline ad 1 oz., which has been of great service. No strong soap should be used nor much water, but a little weak tar lotion may be used with fine oatmeal for bathing the affected parts.

Molluscum Contagiosum and Lichen Planus.

Here is a boy, aged 15, with molluscum contagiosum. The tumours are grouped about his left knee, and are of the size of a pea. A few have, as you can see, undergone suppuration, and in their present state could hardly be distinguished from an involuting impetigo or a pemphigus. However, he has some really typical ones upon the inner side of the joint, and also upon the abdomen. Once seen the condition is not likely to be missed again, the waxy, sessile, umbilicated growths being absolutely characteristic. By pressure with the thumb nails the cheesy content of the tumour can be evacuated, and if this be placed under a microscope in a drop of potash-solution the so-called "molluscum-bodies" can be seen in abundance. Their exact nature has given rise to much discussion. They are not parasites, but merely degenerated epithelial cells containing kerato-hyalin, and of their mildly contagious character there can now be no doubt. When the lesions are very numerous indeed they may give rise to some difficulty in diagnosis, and they have even been mistaken for smallpox. The treatment consists in squeezing out the contents and touching the cavity with pure phenol, a tiny double-edged lancet being a convenient instrument for the purpose.

This is a case of lichen planus hypertrophicus in a Jewess, aged 44. The legs show the condition in a most marked degree, there being purplish coalesced patches raised above the general surface, and very slightly scaly. Some of the primary lesions in the shape of the characteristic plane papules may still be seen, many of them running together to form patches. The disease has lasted for three months, the exciting cause being exposure to cold after a bath. The arms show simply pigmentation and retrogression of plane papules, and you would hardly be expected to make a diagnosis from these parts alone. The mucous membrane of the mouth is unaffected, though some of you may recollect the case of a young man showed at the last demonstration in whom the curious white streaks upon the buccal mucosa were very conspicuous. This patient is responding well to arsenical treatment, and her pruritus, which has been very severe, is kept under control by baths containing a little cyllin.

